# Exploring the use of ChatGPT to analyze student course evaluation comments

**DOI:** 10.1186/s12909-024-05316-2

**Published:** 2024-04-19

**Authors:** Kathryn A. Fuller, Kathryn A. Morbitzer, Jacqueline M. Zeeman, Adam M. Persky, Amanda C. Savage, Jacqueline E. McLaughlin

**Affiliations:** 1 UNC Eshelman School of Pharmacy, University of North Carolina at Chapel Hill, Chapel Hill, NC USA; 2 Center for Innovative Pharmacy Education and Research, UNC Eshelman School of Pharmacy, University of North Carolina at Chapel Hill, Chapel Hill, NC USA

**Keywords:** ChatGPT, OpenAI, Course evaluations, Chatbot, Efficiency, Qualitative analysis

## Abstract

**Background:**

Since the release of ChatGPT, numerous positive applications for this artificial intelligence (AI) tool in higher education have emerged. Faculty can reduce workload by implementing the use of AI. While course evaluations are a common tool used across higher education, the process of identifying useful information from multiple open-ended comments is often time consuming. The purpose of this study was to explore the use of ChatGPT in analyzing course evaluation comments, including the time required to generate themes and the level of agreement between instructor-identified and AI-identified themes.

**Methods:**

Course instructors independently analyzed open-ended student course evaluation comments. Five prompts were provided to guide the coding process. Instructors were asked to note the time required to complete the analysis, the general process they used, and how they felt during their analysis. Student comments were also analyzed through two independent Open-AI ChatGPT user accounts. Thematic analysis was used to analyze the themes generated by instructors and ChatGPT. Percent agreement between the instructor and ChatGPT themes were calculated for each prompt, along with an overall agreement statistic between the instructor and two ChatGPT themes.

**Results:**

There was high agreement between the instructor and ChatGPT results. The highest agreement was for *course-related topics* (range 0.71-0.82) and lowest agreement was for *weaknesses of the course* (range 0.53-0.81). For all prompts except *themes related to student experience*, the two ChatGPT accounts demonstrated higher agreement with one another than with the instructors. On average, instructors took 27.50 ± 15.00 min to analyze their data (range 20–50). The ChatGPT users took 10.50 ± 1.00 min (range 10–12) and 12.50 ± 2.89 min (range 10–15) to analyze the data. In relation to reviewing and analyzing their own open-ended course evaluations, instructors reported feeling anxiety prior to the process, satisfaction during the process, and frustration related to findings.

**Conclusions:**

This study offers valuable insights into the potential of ChatGPT as a tool for analyzing open-ended student course evaluation comments in health professions education. However, it is crucial to ensure ChatGPT is used as a tool to assist with the analysis and to avoid relying solely on its outputs for conclusions.

**Supplementary Information:**

The online version contains supplementary material available at 10.1186/s12909-024-05316-2.

## Background

The release of the large language model (LLM) ChatGPT (chatbot generative pre-trained transformer) caused an immediate change in perspective in medical education [1]. With this generative artificial intelligence (AI) tool capable of generating human-like responses from human prompts, concerns were raised around academic honesty and plagiarism with the use of ChatGPT in classroom and online assessments. These concerns quickly led to several schools in higher education implementing policies and procedures around student use of ChatGPT to ensure protection of assessments and promote honesty and integrity [2]. While there have been some concerns around the development of ChatGPT, a growing number of positive applications in higher education have been found for both students and faculty. There is literature to support the use of ChatGPT in student learning to teach critical thinking and writing skills [3]. Additionally, faculty can reduce workload by implementing the use of AI in rigorous and time consuming tasks such as generating test questions, grading student assessments, and creating clinical scenarios [1].

In parallel with the development of ChatGPT, the field of text analytics (e.g., text mining) has been steadily evolving within higher education [4, 5]. Text mining has been employed to increase faculty efficiency by extracting valuable insights from vast quantities of text-based materials, such as student reflections and preceptor comments [6, 7]. As an example, text mining was used to increase faculty efficiency by identifying students at risk for failing clinical rotations based on 4000 preceptor comments and identifying common topic themes across 7000 student essays [7]. While text mining has shown promise, it remains a labor-intensive process reliant on human judgment to distill relevant information. In contrast, ChatGPT, with its advanced natural language processing capabilities, offers the potential to streamline this process by automatically generating meaningful information without extensive manual intervention [8].

Student course evaluations are a common tool used across higher education that allow faculty to collect student quantitative and qualitative feedback on courses with the ultimate goal of continuous course quality improvement [9]. While faculty in medical education have acknowledged the value of course evaluation for course improvement, the process of identifying useful information with multiple open-ended comments is often time consuming and difficult [9, 10]. One recent study examined the faculty process for reviewing course evaluations and reported that the most common issue identified was the large quantity of course comments received each semester. This also included challenges associated with determining common themes across student feedback and the significant time required to review the large quantity of comments [9].

Prior to the creation and popularization of ChatGPT, several applications using an automated tool based on natural language processing have been in use to provide student feedback to instructors. One early example, Hubert®, was designed as a chatbot that asks students questions about the quality of the class and teaching [11, 12]. The conversational messenger format of the application allowed students to identify strengths and areas of improvement for the course while the chatbot was subsequently organizing and synthesizing the feedback into a report viewable on an online dashboard for the instructor. Strengths and areas for improvement were collated by an AI analysis of repeatedly invoked phrases and sentiments [12]. Similarly, researchers at Stanford developed M-Powering Teachers®, an application designed to provide automated feedback to instructors. The tool demonstrated capability in providing instructors with information on the extent to which they understood a student’s statement and built on that idea during class as well as feedback on the instructor’s questioning practices [13]. Examples such as these demonstrate the ability to use AI to provide instructor specific feedback on teaching practices and reinforce the need to explore modern AI tools further.

The recent release of ChatGPT offers an opportunity for medical educators to consider various ways in which the tool might be leveraged to support their teaching. While some literature exists on the use of ChatGPT to increase efficiencies for academic work in areas such as creating problem-based learning cases, writing examination questions, and developing discussion questions [14–16], there is an overall lack of literature in medical education around how faculty can increase the efficiency of course evaluation review. The purpose of this study was to explore the use of ChatGPT in analyzing course evaluation comments, including the time required to generate themes and the level of agreement between instructor-identified themes and AI-identified themes.

## Methods

In June 2023, four instructors from the University of North Carolina (UNC) Eshelman School of Pharmacy independently analyzed student course evaluation comments for one of their own courses. Five prompts were provided to guide the coding process, such as *“What were 5 strengths of your course from the student perspective?”* In addition, instructors were asked to note the time required to complete the analysis, the general process they used to analyze the student comments, and how they felt during their analysis. Instructors were selected based on the various topics they taught and the varied teaching methods and settings they utilized in the School’s Doctor of Pharmacy (PharmD) degree program.

By request, instructors provided the student open-ended comments when submitting the results of their analysis to the research team. Once the student comments were received, they were provided to two independent Open-AI ChatGPT users for analysis. OpenAI ChatGPT was used as the AI system in this study given its wide availability, ease of use, and LLM engine. Two ChatGPT user accounts were utilized with slightly different prompts to explore variations in how the system might analyze these types of data. All comments were anonymized by the ChatGPT users prior to ChatGPT analysis to protect anonymity of the instructors within ChatGPT.

Thematic analysis was used to analyze the themes generated by instructors and ChatGPT. For each course, results from ChatGPT were coded by three independent researchers using the themes identified by each instructor. In other words, each researcher determined whether each ChatGPT theme aligned with any of the instructor-identified themes for each prompt. Percent agreement between the instructor and each ChatGPT account was calculated for each prompt, along with an overall agreement statistic between the instructor and two ChatGPT accounts. Mean ± standard deviation (SD) was used to describe the data. Results from the ChatGPT analysis were provided back to each instructor for member-checking.

This study was submitted to the University of North Carolina Institutional Review Board (#21–0379) and determined to be not human subjects research. A written description of the project was provided to instructors at the start of the study (e.g., low risk, voluntary, confidentiality, and research contact information) and implied consent was utilized.

## Results

As seen in Table 1, the courses included in this analysis focused on various topics (e.g., foundational math and sciences, professional development, clinical skill development, pharmaceutical science) using varied teaching methods (e.g., flipped models, skills lab) and settings (e.g., large lecture hall, small group learning). A total of 470 (117.50 ± 114.14) comments were analyzed. On average, instructors identified 23.50 ± 5.74 themes and ChatGPT identified 29.75 ± 6.50 themes in response to the 5 prompts. In some cases, multiple ChatGPT-identified themes aligned with a single instructor-identified theme.

Examples of instructor-identified themes and ChatGPT-identified themes can be found in Tables 2 and 3. In one course, for example, the instructor identified “Instructors were engaging and enjoyable” as a *student perspective or experience* while ChatGPT account 1 found “Appreciation for engaging and interactive teaching methods” and “Importance of practice test questions and faculty encouragement” and ChatGPT account 2 found “Appreciated engaging lectures and interactive activities” for the same prompt. In some instances, ChatGPT elaborated more broadly on the theme, whereas the instructor provided a concise and specific finding. For example, in one course, the instructor stated one change identified in the course evaluations was to “end class on time”. Alternatively, ChatGPT account 1 phrased this finding as “Address the concerns regarding the early morning class time and strive to end classes on time. Consider rearranging the schedule or providing more breaks to ensure that class activities fit within the allotted time frame”. Similarly, ChatGPT account 2 stated “Address the concerns raised by students regarding the course going past the scheduled time or feeling dragged on. Consider implementing strategies to manage time more effectively during class sessions, ensuring that topics are covered within the allocated time frames and maintaining an engaging pace throughout the course”. In general, there was high agreement between the instructors and ChatGPT accounts (Table 4). The highest agreement between instructors and the ChatGPTs was for *course-related topics* (range 0.71 to 0.82) and lowest agreement was for *weaknesses of the course* (range 0.53 to 0.81). For all prompts except *themes related to student experience*, the two ChatGPT accounts demonstrated higher agreement with one another than with the instructors.

On average, instructors took 27.50 ± 15.00 min to analyze their data (range 20–50 min). The ChatGPT users took 10.50 ± 1.00 min (range 10–12 min) and 12.50 ± 2.89 min (range 10–15 min) to analyze the data, which included formatting and anonymizing the data. When asked about the process used and emotions experienced when reading and analyzing their course evaluations, all instructors (*n* = 4, 100%) described the use of an iterative analysis process, reading comments at least 2 times to identify themes and notable feedback. Instructors reported feeling some anxiety prior to the process of reviewing their own open-ended course evaluations (e.g., “stress”, “fear of the unknown”, “anxious”), satisfaction during the process (e.g., “satisfied with constructive recommendations”, “joy”, “relief”), and frustration related to findings (e.g., “things didn’t go as well as intended”, “frustrated that students didn’t find value in [activity]”).

**Table 1:** Course Characteristics

Course Focus	Course Setting	Course Pedagogy	Number of Student Comments Analyzed	Number of Instructor-Identified Themes	Number of ChatGPT-Identified Themes
Pharmaceutical Science	Large lecture-style classroom	Flipped (i.e., pre-class learning & in-class activities)	39	16	21
Professional Development	Large lecture-style classroom	Mix of lecture and small group activities	57	24	33
Skills Development	Small groups with instructor	Lab-based with OSCE assessments	88	30	36
Foundational Math & Science (i.e., Pre-Requisite Topics)	Large lecture-style classroom	Flipped (i.e., pre-class learning & in-class activities)	286	24	29

OSCE = Objective Structured Clinical Examination

**Table 2:** Examples of Course Strengths Identified by Instructors and Related ChatGPT Findings

Example Strength (I): What were 5 strengths of your course from the student perspective?	Example Strength (Chat1): What were 5 strengths of my course from the student perspective?	Example Strength (Chat2): I am a professor at a pharmacy school teaching a [Course Title] course. What were the 5 strengths of this course from the student perspective?
Focused on learning	Students appreciated your ability to explain topics clearly, use relevant examples, and provide practice opportunities that facilitated their learning.	The professor was highly praised for their teaching style, explanations, and use of relevant examples. Students appreciated how the material was presented in a way that facilitated their learning.
Technology-free zone activities	Students appreciated designated times without technology and enjoyed engaging in drawing exercises, which raised the mood and created a positive learning environment.	Students appreciated the interactive nature of the course, including team-based activities, technology-free zones, and drawing exercises. These activities helped to reinforce the course content and promote active learning.
Hands on learning	Students valued the hands-on learning experiences in the course, particularly the opportunities to practice patient interactions and work with medical devices. They felt that these experiences improved their confidence and preparedness for real-world patient care.	The course provides hands-on experience and practical skills that are directly applicable to patient care, preparing students for real-world interactions.
Course structure– pre-class learning with in-class activities	The course was praised for its clear expectations and well-structured organization. Students found the course to be well-paced, with clear module information and building blocks of content, which helped them stay on track and understand the material effectively.	Students praised the organization and structure of the course, with clear learning objectives and expectations. The course’s design allowed students to focus on key topics and understand the course load for future classes.
*No theme identified about instructor flexibility and kindness*	Flexibility and kindness towards students	Flexibility and understanding towards students’ needs and stress levels

I = instructor; Chat1 = ChatGPT account 1; Chat2 = ChatGPT account 2

**Table 3:** Examples of Course Changes Identified by Instructors and Related ChatGPT Findings

Example Change (I): Please list 3–4 changes you might make to your course or teaching based on the course evaluation comments:	Example Change (Chat1): Please list 3–4 changes I might need to make to my course or teaching based on the course evaluation comments.	Example Change (Chat2): I am a professor at a pharmacy school teaching a [Course Title] course. Please list 3–4 changes you might make to your course or teaching based on the course evaluation comments:
End class on time	Address the concerns regarding the early morning class time and strive to end classes on time. Consider rearranging the schedule or providing more breaks to ensure that class activities fit within the allotted time frame.	Address the concerns raised by students regarding the course going past the scheduled time or feeling dragged on. Consider implementing strategies to manage time more effectively during class sessions, ensuring that topics are covered within the allocated time frames and maintaining an engaging pace throughout the course.
Provide more practice problems	Students expressed a desire for more interactive and hands-on learning opportunities. Incorporating more group work, class discussions, and hands-on activities can foster student engagement and deeper understanding of the course material. Consider integrating activities that encourage active participation and collaboration among students.	Identify [topics] where students have expressed a desire for more practice questions. Provide additional practice resources to reinforce their understanding of these challenging topics and support their learning process.
Provide more structure for lab groups and TAs	Address the feedback regarding the lack of clarity and structure in certain aspects of the course. Take steps to provide clearer instructions, guidelines, and expectations for assignments and activities. Consider establishing a more structured framework or schedule to ensure students have a clear understanding of what they should be doing and when.	Address the concerns regarding the lack of clarity and structure by providing a detailed course outline and schedule at the beginning of the semester. Ensure timely and clear communication of important information, such as OSCE details and assignments, by providing advance notice to students, preferably a week ahead, to allow better preparation and time management.
*No theme identified about communication*	Several students mentioned the need for clearer communication, especially in answer keys and instructions. Take the feedback into account and work on providing more comprehensive and detailed explanations. Ensuring clarity in communication will help students better understand expectations and improve their overall learning experience.	Improve communication and clarity. Address feedback related to the clarity of instruction by refining explanations and ensuring that key concepts are thoroughly covered. Consider offering additional examples and explanations to help students better grasp complex topics.
*No theme identified about assessment alignment*	Students expressed a desire for better alignment between the homework assignments and quiz questions. Review the content and format of the homework assignments to ensure they directly prepare students for the quiz questions and help them succeed.	To address the concern about the disconnect between homework assignments and quizzes, review and revise the homework assignments to ensure they align closely with the quiz content. This will help students see a direct connection between their practice and the assessments.

I = instructor; Chat1 = ChatGPT account 1; Chat2 = ChatGPT account 2

**Table 4:** Agreement between Instructors and ChatGPT

Prompt Focus	Agreement– M ± SD			*All*
	*I/Chat1*	*I/Chat2*	*Chat1/Chat2*	
**Course-related Topics**	0.80 ± 0.17	0.71 ± 0.14	0.82 ± 0.14	0.89 ± 0.04
**Themes related to Student Experience**	0.82 ± 0.14	0.60 ± 0.19	0.73 ± 0.13	0.85 ± 0.08
**Strengths of the Course**	0.68 ± 0.11	0.71 ± 0.12	0.92 ± 0.17	0.88 ± 0.06
**Weaknesses of the Course**	0.53 ± 0.06	0.57 ± 0.08	0.81 ± 0.24	0.80 ± 0.04
**Changes You Might Make**	0.71 ± 0.28	0.58 ± 0.29	0.89 ± 0.13	0.86 ± 0.10

I = instructor; Chat1 = ChatGPT account 1; Chat2 = ChatGPT account 2; M = Mean; SD = Standard Deviation

## Discussion

The emergence of generative AI tools, exemplified by ChatGPT, is transforming the landscape of higher education, including the health professions [1, 3, 14]. However, there is no known research examining the use of ChatGPT to assess student feedback provided through course evaluations. This study aligns with recent research highlighting common challenges faced by faculty around managing the large volume of course evaluation comments and identifying common themes [9, 17, 18]. Findings from this study indicated that ChatGPT was able to generate themes from student course evaluation comments that agreed with those generated by instructors for most course-related items. Notably, ChatGPT identified a higher number of themes in a shorter period of time and often provided more depth compared to themes generated by instructor manual review.

Overall, notable levels of agreement between instructors and ChatGPT were found across a diverse range of courses, teaching topics, methods, and settings. The congruence between thematic analysis by humans and a LLM tool found in this study was comparable to previous literature in the health professions evaluating the use of LLM tools on qualitative data responses. In one study comparing the level of agreement of experiential preceptor comments between faculty coders and a sentiment analysis performed by a LLM tool, agreement was found to be > 90% [6]. Similarly, sentiment analysis via a machine learning process from free text has been shown to provide a reasonably accurate assessment (> 80% agreement) of patients’ opinion about different performance aspects of a hospital [19]. These findings, along with the results of this study, suggest that a LLM tool shows promise as a way to automate analysis of text. Additionally, the work of this study expands on previous literature by comparing the time required to analyze the data with the LLM, ChatGPT, to human analyzers. This study found that anonymizing the data, formatting for submission, and analyzing via ChatGPT required less than half the time instructors required to analyze the data. This suggests that ChatGPT can effectively assist in the thematic analysis of student comments and streamline the process all while potentially reducing the burden on faculty members.

One key aspect to highlight is that ChatGPT identified more themes, on average, than the faculty themselves, as well as provided more depth and detail of the themes. For example, the instructor may have identified one suggested change, whereas both ChatGPT accounts identified the suggested change along with potential solutions, suggesting that ChatGPT has the potential to provide a more comprehensive analysis of student feedback. However, it is important to note that in some cases, multiple ChatGPT-identified themes aligned with a single instructor-identified theme. This may reflect the granularity of analysis that ChatGPT can achieve, but it also raises questions about the relevance and utility of some of the additional themes identified by using AI. Therefore, it appears to still be necessary for faculty to exercise judgment and discretion when using ChatGPT for thematic analysis and carefully review the generated themes to ensure their relevance to the specific context.

The highest agreement between faculty and ChatGPT accounts was observed for course-related topics, indicating that ChatGPT is reasonably comparable to human analysis at capturing feedback related to the course content, teaching methods, and overall course experience. In contrast, the lowest agreement was found for weaknesses of the course. The lower level of agreement may be related to the wide range of emotions experienced during the course evaluation analysis process that faculty members reported, such as anxiety, satisfaction, and frustration. These emotions highlight the personal and often subjective nature of analyzing student feedback. ChatGPT, as an AI tool, does not have the ability to “feel” the impact of a negative comment and should not be influenced by emotional factors, which may therefore lead to less biased analysis [20]. This point may explain the lower level of agreement in identifying weaknesses within course evaluations, as the instructor’s emotions and biases related to a course may influence their ability to evaluate critical feedback. Conversely, the lower level of agreement may suggest that ChatGPT missed certain nuances in student comments that faculty may inherently understand. Similar to above, this may indicate that faculty should rely on their expertise and context of the course to interpret and address areas of improvement highlighted by students. As a next step, analysis of course evaluation comments by educators not associated with the course might provide insight into the nature and source of this lower level of agreement.

Efficiency was another critical aspect in assessing the use of ChatGPT to analyze student feedback via course evaluations. Instructors in this study reported spending a substantial amount of time analyzing student comments, with an average of 27.5 min per course. In contrast, ChatGPT users completed the analysis in less time, with an average of 10.5 and 12.5 min for the two accounts, respectively. This time-savings can be exponential for instructors with multiple course evaluations from different teaching activities, and also for units (e.g., curriculum committee, assessment committee, assessment offices, curriculum leadership) responsible for reviewing course evaluation results for an entire program and/or curriculum.

While this study demonstrated several positive aspects of using ChatGPT to analyze course evaluation feedback, there are several important limitations to consider when using ChatGPT for any type of qualitative analysis [21–23]. In particular, cleaning the data and creating effective prompts are essential steps to enhance the quality and relevance of ChatGPT-generated content for qualitative analysis. Additionally, there may be constraints around the quantity of text that some versions of ChatGPT can analyze at one time. Therefore, this constraint could result in the user needing to break up the text into a smaller and more manageable quantity. These steps can add additional time to the task, reducing the efficiency seen above with ChatGPT analyzing student comments. As discussed previously, ChatGPT also lacks contextual understanding and critical thinking abilities. This limitation means that it is essential for users of ChatGPT to provide sufficient context to guide the model’s responses and to review the generated text to ensure that the content aligns with the desired context and meaning. While ChatGPT has the potential to provide a more objective approach, that does not mean that ChatGPT generated responses are without their own biases. ChatGPT, and similar AI language models, incorporate biases from the data they are trained on, as they learn patterns and associations from the vast amounts of text data collected from the internet [24]. These limitations highlight that with any type of qualitative analysis, including that which was performed in this study, it’s essential to use ChatGPT as a tool to assist with the analysis and to avoid relying solely on its outputs.

There are also several limitations specific to this study that are important to note. The study involved a limited sample size, using four instructors and four courses from a single institution. While a reduced sample size was targeted given the exploratory nature of this study, it may not be representative of the broader population of instructors and courses within health professions education; however, this work was designed to demonstrate a generalizable technique for analysis and not designed to generate generalizable results (i.e., course evaluation themes). Additionally, two different ChatGPT accounts with slightly different prompts were used and their responses were compared. The variations between these accounts may have affected the results produced, however, this limitation was mitigated by having the users generate the ChatGPT responses on the same day at approximately the same time. The analysis showed the highest agreement among the two ChatGPT accounts for most prompts, suggesting relative consistency in their analyses. Despite these limitations, this study sheds light on the potential of ChatGPT as a valuable tool in the analysis of student course evaluation feedback in health professions education.

## Conclusions

This study offers valuable insights into the potential of ChatGPT as a tool for analyzing open-ended student course evaluation comments in health professions education. It demonstrated a high level of agreement between most instructor-identified themes and ChatGPT-identified themes. Moreover, ChatGPT reduced the time required for analysis, potentially easing the burden on course instructors and provided more detail to the identified themes. However, it is crucial to use ChatGPT judiciously, as it may generate additional unnecessary information and/or miss themes that require validation and context-specific interpretation. Future research should explore how prompt language may impact the themes yielded and the integration of ChatGPT into health profession education program workflows to further assess its impact on course quality improvement and faculty workload. As AI technologies continue to evolve, their role in education, particularly in the context of feedback analysis, is likely to expand and become increasingly valuable.

### Electronic supplementary material

Below is the link to the electronic supplementary material.

Supplementary Material 1

## Data Availability

The datasets generated and analyzed during the current study are not publicly available to protect the integrity of the courses included in this study but are available from the corresponding author upon reasonable request.
