# Supplementary material for: Exploring the use of ChatGPT to analyze student course evaluation comments
Source: BMC Med Educ. 2024 Apr 19;24:423. doi: 10.1186/s12909-024-05316-2 (PMC11031883; doi:10.1186/s12909-024-05316-2)
Supplement: Supplementary file 1 — Supplementary Material 1 [file 12909_2024_5316_MOESM1_ESM.docx]

**Supplementary Table.** Prompts used by instructors and ChatGPT users to identify themes in course evaluation comments from students.

| **Instructor Prompts** | **ChatGPT1 Prompts** | **ChatGPT2 Prompts** |
| --- | --- | --- |
| Please list up to 10 course-related topics you identified in the course evaluation comments below. | I am an instructor teaching [Course Title] at a University. Today I would like your assistance to examine the student evaluation of the course and instructor. Please list up to 10 course-related topics you identified in the course evaluation comments below. | I am a professor at a pharmacy school teaching a [Course Title] course. Please list up to 10 course-related topics you identified in the course evaluation comments below. |
| Please list up to 10 themes related to student perspectives or experiences below. | Please list up to 10 themes related to student perspectives or experiences below. | I am a professor at a pharmacy school teaching a [Course Title]. Please list up to 10 themes related to student perspectives or experiences below using the comments. |
| What were 5 strengths of your course from the student perspective? | What were 5 strengths of my course from the student perspective? | I am a professor at a pharmacy school teaching a [Course Title] course. What were the 5 strengths of this course from the student perspective? |
| What were 5 weaknesses of your course from the student perspective? | What were 5 weaknesses of my course from the student perspective? | I am a professor at a pharmacy school teaching a [Course Title] course. What were the 5 strengths of this course from the student perspective? |
| Please list 3-4 changes you might make to your course or teaching based on the course evaluation comments. | Please list 3-4 changes I might need to make to my course or teaching based on the course evaluation comments. | I am a professor at a pharmacy school teaching a [Course Title] course. Please list 3-4 changes you might make to your course or teaching based on the course evaluation comments. |
